# Relationships of weight perceptions with weight control related behaviors among Chinese children and adolescents: A school-based study in Zhejiang Province

**DOI:** 10.1371/journal.pone.0285205

**Published:** 2023-05-17

**Authors:** Zhu Yu, Guanping Dong, Wei Wu, Ke Huang, Xiao-Yan Zhou, Hao Wang, Meng Wang, Junfen Fu

**Affiliations:** 1 Department of Endocrinology, Children’s Hospital Zhejiang University School of Medicine, National Clinical Research Center for Child Health, National Children’s Regional Medical Center, Hangzhou, China; 2 Zhejiang Provincial Center for Disease Control and Prevention, Hangzhou, China; Holy Spirit University of Kaslik: Universite Saint-Esprit de Kaslik, LEBANON

## Abstract

**Objectives:**

Weight perceptions have been implicated in weight control related behaviors among children and adolescents, yet studies in mainland China are scarce. We examined the associations of self-perceived weight status and weight misperception with weight control related behaviors in Chinese middle and high school students.

**Methods:**

We used cross-sectional data from the 2017 Zhejiang Youth Risk Behavior Survey which that included 17,359 Chinese students, with 8,616 boys and 8,743 girls. Perceived weight status, as well as height, weight and weight control related behaviors information was collected via a self-reported questionnaire. Odds ratios (ORs) with 95% confidence intervals (CIs) calculated by multinomial logistic regression were used to assess the relationships between weight perceptions and weight control related behaviors.

**Results:**

Among the 17,359 students aged 9 to 18 years, the mean (SD) age was 15.72 (1.64) years. Overall, 34.19% of children and adolescents perceived themselves as overweight and the prevalence of weight misperception was 45.44%, with 35.54% overestimation and 9.90% underestimation. Children and adolescents perceiving themselves as overweight were more likely to have weight control behaviors, with OR was 2.60 (95% CI: 2.39–2.83) for weight control attempt, 2.48 (2.28–2.70) for exercising, 2.85 (2.60–3.11) for dieting, 2.01 (1.51–2.68) for taking laxatives, 2.09 (1.67–2.02) for taking diet pills, and 2.39 (1.94–2.94) for fasting, respectively, compared to those with right weight status. Among children and adolescents with overestimating weight status, the OR was 2.40 (2.22–2.59), 2.50 (2.31–2.70), 2.85 (2.61–3.11), 1.81 (1.39–2.37), 2.20 (1.77–2.74), and 2.16 (1.77–2.63) for weight control attempt, exercising, dieting, taking laxatives, taking diet pills, and fasting, relative to those with accurate weight perception.

**Conclusions:**

Self-perceived overweight and weight misperception are prevalent in Chinese children and adolescents, and positively associated with weight control related behaviors.

## Introduction

Obesity in children and adolescents remains a major public health concern worldwide. Recent estimates showed the global prevalence of obesity among girls increased from 0.7% to 5.6% and from 0.9% to 7.8% among boys during 1975–2016, with the number of obese individuals has reached 50 and 74 million, respectively [1]. Past decades have witnessed substantial growth of obesity in China, meanwhile, data from the latest national survey suggested that 7.9% of Chinese children and adolescents aged 6 to 17 years were estimated to be obese in 2015–19 [2]. Based on prior knowledge, obesity occurred in childhood and adolescence can not only lead to the short-term psychological consequences, such as depression, lower self-esteem and emotional disorders [3], but also increase the risk of long-term adverse health outcomes, including the cardiometabolic disease and certain types of cancer in adulthood [4]. The obesity epidemic and potential negative impacts on health highlight the need to research on weight control related behaviors at early stages of life.

Adolescence is a transitional stage with many psychological changes taking place. As a primary dimension of body image central to emotional well-being, weight perceptions play an important role in body management [5]. Previous studies have suggested that self-perceived weight status was a stronger predictor of weight control effort and practices than the actual weight status [6, 7]. Evidence from a recent systematic review confirmed that perceived overweight was associated with weight loss attempts, as well as weight control strategies [8]. Besides, researchers found that children and adolescents tended to misperceive their weight status [9]. According to separate national survey, prevalence of weight misperception among adolescents in the United States and South Korea was estimated to be 42.1% (35.3% underestimation and 6.8% overestimation) and 50.2% (23.4% underestimation and 26.8% overestimation), respectively [10, 11]. Furthermore, as a prevalent phenomenon in youth, increasing literature have linked the weight misperception with unhealthy weight control behaviors, such as fasting, laxative use and taking diet pills [12, 13]. By contrast, there is little information on weight perceptions and their associations with weight control related behaviors among Chinese children and adolescents. Particularly, although a few surveys have indicated the weight misperception was also obvious among Chinese youth, these studies mainly focused on outcomes of dietary and exercise patterns, not exclusively the weight control related behaviors [14, 15].

Therefore, the primary objectives of this study were to describe the self-perceived weight status and weight misperception among Chinese school-aged children and adolescents, and to explore their possible associations with weight control related behaviors.

## Materials and methods

### Study design and participants

The Zhejiang Youth Risk Behavior Survey (YRBS) is an ongoing school-based study conducted by Zhejiang Provincial Center for Disease Control and Prevention (CDC). The YRBS study is designed to assess the health-related behaviors in Chinese school-aged children and adolescents of Zhejiang Province. Details on the study design and participants have previously described elsewhere [16]. Briefly, the current analysis data were from the latest survey conducted in 2017, in which multistage, stratified cluster sampling methods were used and 23,554 Chinese students were successfully recruited. In the first stage, 30 counties (12 urban and 18 rural areas) were sampled from all 90 counties of Zhejiang Province on the basis of socioeconomic status. Then, all schools in selected counties were stratified according to their levels (middle school, academic and vocational high school) and geographical positions (from west to east, from north to south). In the second stage, based on the number of students in each level of school, samples of classes were chosen in each level of school. In the third stage, students in all selected classes were invited to participate in the survey. After excluding the participants with non-local census register and those with missing key information on age, as well as gender, and self-reported height and weight, 17,359 students aged 9 to 18 years including 8,616 boys and 8,743 girls were finally included in the present study. Self-reported information on demographic characteristics, smoking, alcohol drinking, dietary intake, physical activity, sleep duration, weight control attempt and strategies, injury, and other health-related behaviors was collected via a self-administered questionnaire. The questionnaire used was derived from US 1991–2015 Youth Risk Behavior Surveillance System (YRBSS) and Global School-based Student Health Survey (GSHS). The students completed the anonymous questionnaire in the classroom independently, and afterward, questionnaires were collected by the researchers. To make all the participants voluntary, parents / guardians of the students and the school officials were sent a written letter and given the option to refuse. Besides, all the researchers were strictly trained to protect the students’ privacy and ensure the confidentiality of the personal data. In particular, this study abided by the “Declaration of Helsinki” and was approved by the ethics committee of Zhejiang CDC.

### Assessment of exposure variables

In this survey, self-perceived weight status was assessed by the following question, “How do you describe your weight?” with response options as follows, very underweight, slightly underweight, about the right weight, slightly overweight, and very overweight. Particularly, participants who reported “very” or “slightly” underweight were categorized as “underweight” and those that reported “very” or “slightly” overweight were categorized as “overweight”.

The accuracy of weight perceptions were classified as overestimated, underestimated and accurate by comparing the self-perceived and the actual weight status based on body mass index (BMI, underweight, normal weight, overweight, obesity). BMI was categorized using the age-and sex-specific cut-off points developed by the Childhood Obesity Working Group of the International Obesity Task Force (IOTF) [17, 18]. To make the comparisons between the self-perceived and actual weight status, we combined the specific BMI groups of overweight and obesity into a single category (overweight / obesity). Weight perception of overestimated included those underweight children and adolescents who perceived themselves as about the right weight or overweight and normal weight children and adolescents who perceived as overweight. Underestimated included normal weight children and adolescents who perceived themselves as underweight and overweight / obesity children and adolescents who perceived as about the right weight or underweight. Weight perception of neither overestimated nor underestimated was defined as the accurate.

### Assessment of outcome variables

Trying to control weight was determined by asking “Are you doing something to lose or to keep from gaining weight?”.

According to prior knowledge [19, 20], exercising was considered as a healthy weight control behavior, which was assessed by the following question, “During the past two years, did you exercise to lose or to keep from gaining weight?”. Unhealthy weight control behaviors that included dieting, taking laxatives, taking diet pills, and fasting were assessed with the following questions, respectively, “During the past two years, did you eat less food or few calories to lose or to keep from gaining weight?”, “During the past two years, did you take laxatives to lose or to keep from gaining weight?”, “During the past two years, did you take diet pills without a doctor’s advice to lose or to keep from gaining weight?”, “During the past two years, did you go without eating for 24 hours or more to lose or to keep from gaining weight?”. The above weight control related behaviors were all dichotomized with a “yes” or “no” response.

### Other covariates

Socio-demographic characteristics and some lifestyle behaviors potentially associated with weight control related behaviors were also considered in this analysis. They were age (≤13, 14, 15, ≥16 years), gender (boys, girls), location of school (rural, urban), paternal education (middle school or below, high school, college or above), maternal education (middle school or below, high school, college or above), BMI (underweight, normal weight, overweight / obesity), current smoking (yes, no), breakfast consumption (daily, non-daily), and muscle strengthening activity (yes, no). Among them, current smoking was assessed by the following question, “During the past 30 days, on how many days did you smoke cigarettes? (0 day, 1–2 days, 3–5 days, 6–9 days, 10–19 days, 20–29 days, 30 days)”. Participants who had smoked at least 1 day during the past 30 days were considered to have the current smoking behavior. Breakfast consumption and muscle strengthening activity were assessed with the following questions, respectively, “During the past 7 days, how many days did you eat breakfast? (0 day, 1 day, 2 days, 3 days, 4 days, 5 days, 6 days, 7 days)”, “During the past 7days, how many days did you do exercises to strengthen or tone your muscles, such as push-ups, sit-ups, or weight lifting, etc.? (0 day, 1 day, 2 days, 3 days, 4 days, 5 days, 6 days, 7 days)”.

### Statistical analysis

Descriptive statistics were used to describe the distributions of self-perceived weight status and accuracy of weight perceptions among children and adolescents with different characteristics, which were compared using the χ^2^-test. Multinomial logistic regression models were used to estimate the odds ratios (ORs) with 95% confidence intervals (CIs) for the associations between self-perceived weight status, accuracy of weight perceptions and the weight control related behaviors. Adjustment for confounding factors were conducted in three sequential models. In model 1, socio-demographic characteristics of age, gender, location of school, paternal education and maternal education were adjusted. Model 2 was further adjusted for lifestyle behaviors of current smoking, breakfast consumption and muscle strengthening activity. Model 3 was adjusted for all variables in model 2 plus BMI. Besides, on the basis of model 3, associations between self-perceived weight status, weight perceptions and the weight control related behaviors were compared within subgroups defined by gender, location of school and BMI. All analyses were performed using SAS statistical package (version 9.4, SAS Institute, Inc., Cary, NC, USA). All statistical tests were based on the two-sided 5% level of significance.

## Results

### Characteristics of study participants

Among the 17,359 participants aged 9 to 18 years included in analysis, the mean (SD) age was 15.72 (1.64) years. 49.63% of children and adolescents were boys and 39.13% were from urban area. 66.82% of children and adolescents were estimated to be within the normal BMI range, while 24.34% were classified as underweight, and 8.84% were classified as overweight or obese.

Table 1 showed the distributions of self-perceived weight status and accuracy of weight perceptions by socio-demographic characteristics and weight control related behaviors. Overall, 23.01% of children and adolescents perceived themselves as underweight, 42.80% as about right, and 34.19% as overweight. Children and adolescents who perceived themselves as overweight tended to be older, girls, from urban area, with higher BMI, and to try to control weight and engage in specific behaviors (*P* <0.05 for all). Notably, among normal weight children and adolescents, 37.73% perceived themselves as overweight. In comparison with the actual weight status, the total prevalence of weight misperception was 45.44%, with 35.54% overestimation and 9.90% underestimation. Children and adolescents with weight misperception were more likely to be older, girls, from rural area, with lower paternal and maternal education, and to try to control weight and engage in specific behaviors (*P* <0.05 for all). Particularly, overestimation was more common in girls (48.42%) than in boys (22.48%), while underestimation was the dominant misperception in boys (15.53% vs. 4.35%). The prevalence of misperception was higher in underweight children and adolescents (42.47%) and was highest in normal weight children and adolescents (50.46%).

**Table 1:** Distributions of self-perceived weight status and accuracy of weight perceptions by socio-demographic characteristics and weight control related behaviors among children and adolescents in Zhejiang Province.

Characteristics	Self-perceived weight status Number (%)			*P*	Accuracy of weight perceptions Number (%)			*P*
	Underweight	About right	Overweight		Accurate	Underestimated	Overestimated	
Total	3993 (23.01)	7426 (42.80)	5932 (34.19)		9466 (54.56)	1718 (9.90)	6167 (35.54)	
Age groups (years)				<0.001				<0.001
≤13	496 (24.84)	875 (43.82)	626 (31.35)		1117 (55.93)	295 (14.77)	585 (29.29)	
14-	570 (21.23)	1196 (44.54)	919 (34.23)		1451 (54.04)	310 (11.55)	924 (34.41)	
15-	714 (23.09)	1372 (44.37)	1006 (32.54)		1748 (56.53)	311 (10.06)	1033 (33.41)	
≥16	2213 (23.11)	3983 (41.59)	3381 (35.30)		5150 (53.77)	802 (8.37)	3625 (37.85)	
Gender				<0.001				<0.001
Boys	2627 (30.50)	3660 (42.49)	2326 (27.01)		5339 (61.99)	1338 (15.53)	1936 (22.48)	
Girls	1336 (15.63)	3766 (42.10)	3606 (41.27)		4127 (47.23)	380 (4.35)	4231 (48.42)	
Location of school				0.019				0.130
Rural	2471 (23.40)	4564 (43.22)	3526 (33.39)		5697 (53.94)	1059 (10.03)	3805 (36.03)	
Urban	1522 (22.42)	2862 (42.15)	2406 (35.43)		3769 (55.51)	659 (9.71)	2362 (34.79)	
Paternal education				0.714				0.004
Middle school or below	2250 (22.96)	4181 (42.67)	3368 (34.37)		5248 (53.56)	984 (10.04)	3567 (36.40)	
High school	927 (23.49)	1679 (42.55)	1340 (33.96)		2219 (56.23)	365 (9.25)	1362 (34.52)	
College or above	578 (22.85)	1112 (43.95)	840 (33.20)		1445 (57.11)	243 (9.60)	842 (33.28)	
Maternal education				0.419				0.013
Middle school or below	2398 (22.63)	4565 (43.09)	3632 (34.28)		5703 (53.83)	1041 (9.83)	3851 (36.35)	
High school	789 (23.17)	1445 (42.44)	1171 (34.39)		1898 (55.74)	321 (9.43)	1186 (34.83)	
College or above	535 (24.34)	942 (42.86)	721 (32.80)		1256 (57.14)	223 (10.15)	719 (32.71)	
Body mass index (BMI)				<0.001				NA
Underweight	2429 (57.53)	1528 (36.19)	265 (6.28)		2429 (57.53)	NA	1793 (42.47)	
Normal weight	1476 (12.73)	5744 (49.54)	4374 (37.73)		5744 (49.54)	1476 (12.73)	4374 (37.73)	
Overweight / obesity	88 (5.73)	154 (10.03)	1293 (84.23)		1293 (84.23)	242 (15.77)	NA	
Trying to control weight				<0.001				<0.001
Yes	1209 (12.68)	3847 (40.35)	4479 (46.97)		4814 (50.49)	594 (6.23)	4127 (43.28)	
No	2783 (35.64)	3575 (45.78)	1451 (18.58)		4649 (59.53)	1122 (14.37)	2038 (26.10)	
Healthy weight control behavior								
Exercising				<0.001				<0.001
Yes	837 (9.66)	3584 (41.36)	4245 (48.98)		4366 (50.38)	476 (5.49)	3824 (44.13)	
No	3156 (36.35)	3841 (44.24)	1686 (19.42)		5099 (58.72)	1242 (14.30)	2342 (26.97)	
Unhealthy weight control behaviors								
Dieting				<0.001				<0.001
Yes	280 (6.28)	1472 (33.00)	2708 (60.72)		1943 (43.57)	160 (3.59)	2357 (52.85)	
No	3713 (28.80)	5954 (46.19)	3224 (25.01)		7523 (58.36)	1558 (12.09)	3810 (29.56)	
Taking laxatives				<0.001				<0.001
Yes	89 (25.50)	92 (26.36)	168 (48.14)		138 (39.54)	54 (15.47)	157 (44.99)	
No	3904 (22.96)	7334 (43.14)	5763 (33.90)		9328 (54.87)	1664 (9.79)	6009 (35.34)	
Taking diet pills				<0.001				<0.001
Yes	71 (14.06)	145 (28.71)	289 (57.23)		195 (38.61)	43 (8.51)	267 (52.87)	
No	3922 (23.28)	7281 (43.22)	5643 (33.50)		9271 (55.03)	1675 (9.94)	5900 (35.02)	
Fasting				<0.001				<0.001
Yes	108 (16.82)	174 (27.10)	360 (56.07)		255 (39.72)	65 (10.12)	322 (50.16)	
No	3885 (23.25)	7252 (43.40)	5572 (33.25)		9211 (55.13)	1653 (9.89)	5845 (34.98)	

Associations between self-perceived weight status, accuracy of weight perceptions and weight control related behaviors

After adjustment for potential confounders, including socio-demographic characteristics, lifestyle behaviors and BMI, children and adolescents who perceived as overweight were more likely to try to control weight and engage in specific behaviors, compared to those perceiving themselves as about the right weight status. The OR with 95% CI was 2.60 (2.39–2.83) for weight control attempt, 2.48 (2.28–2.70) for exercising, 2.85 (2.60–3.11) for dieting, 2.01 (1.51–2.68) for taking laxatives, 2.09 (1.67–2.02) for taking diet pills, and 2.39 (1.94–2.94) for fasting, respectively. As for children and adolescents who perceived themselves as underweight, positive association was only observed for taking laxatives and the OR was 2.21 (1.57–3.12). Relative to children and adolescents with accurate weight perception, those who overestimated their weight status had increased odds of trying to control weight and engaging in weight control behaviors. The OR was 2.40 (2.22–2.59) for weight control attempt, 2.50 (2.31–2.70) for exercising, 2.85 (2.61–3.11) for dieting, 1.81 (1.39–2.37) for taking laxatives, 2.20 (1.77–2.74) for taking diet pills, and 2.16 (1.77–2.63) for fasting, respectively. Regarding those who underestimated their weight status, similar association was observed for taking laxatives, taking diet pills, and fasting, with the OR was 2.59 (1.83–3.68), 1.50 (1.05–2.13), and 1.49 (1.10–2.02), respectively (Table 2). Stratified by gender, location of school and BMI, most positive associations between weight perceptions and weight control related behaviors were confirmed although some heterogeneity within subgroups was observed (S1 and S2 Tables in S1 File).

**Table 2:** Adjusted odds ratios (95% CIs) of weight control related behaviors by self-perception weight status and accuracy of weight perceptions.

Outcomes	Self-perception weight status	Model 1	Model 2	Model 3	Weight perceptions	Model 1	Model 2	Model 3
Trying to control weight	About right	1.00	1.00	1.00	Accurate	1.00	1.00	1.00
	Underweight	0.40 (0.37–0.44)	0.41 (0.38–0.45)	0.45 (0.41–0.50)	Underestimated	0.52 (0.46–0.58)	0.52 (0.47–0.59)	0.39 (0.35–0.44)
	Overweight	2.85 (2.63–3.08)	2.95 (2.72–3.20)	2.60 (2.39–2.83)	Overestimated	1.91 (1.78–2.05)	1.93 (1.80–2.08)	2.40 (2.22–2.59)
Healthy weight control behavior								
Exercising	About right	1.00	1.00	1.00	Accurate	1.00	1.00	1.00
	Underweight	0.29 (0.26–0.31)	0.29 (0.26–0.32)	0.36 (0.32–0.40)	Underestimated	0.46 (0.41–0.52)	0.47 (0.41–0.53)	0.33 (0.29–0.37)
	Overweight	2.73 (2.53–2.95)	2.94 (2.17–3.18)	2.48 (2.28–2.70)	Overestimated	1.87 (1.74–2.00)	1.90 (1.77–2.05)	2.50 (2.31–2.70)
Unhealthy weight control behaviors								
Dieting	About right	1.00	1.00	1.00	Accurate	1.00	1.00	1.00
	Underweight	0.33 (0.28–0.38)	0.32 (0.28–0.37)	0.39 (0.34–0.46)	Underestimated	0.49 (0.41–0.59)	0.49 (0.41–0.58)	0.38 (0.31–0.45)
	Overweight	3.32 (3.06–3.61)	3.35 (3.09–3.64)	2.85 (2.60–3.11)	Overestimated	2.05 (1.89–2.21)	2.05 (1.89–2.22)	2.85 (2.61–3.11)
Taking laxatives	About right	1.00	1.00	1.00	Accurate	1.00	1.00	1.00
	Underweight	2.02 (1.48–2.76)	1.87 (1.37–2.57)	2.21 (1.57–3.12)	Underestimated	2.68 (1.91–3.77)	2.68 (1.90–3.79)	2.59 (1.83–3.68)
	Overweight	2.26 (1.72–2.97)	2.17 (1.65–2.85)	2.01 (1.51–2.68)	Overestimated	1.66 (1.29–2.14)	1.68 (1.30–2.16)	1.81 (1.39–2.37)
Taking diet pills	About right	1.00	1.00	1.00	Accurate	1.00	1.00	1.00
	Underweight	1.03 (0.76–1.39)	0.96 (0.71–1.31)	1.16 (0.84–1.62)	Underestimated	1.71 (1.21–2.42)	1.70 (1.20–2.41)	1.50 (1.05–2.13)
	Overweight	2.40 (1.94–2.96)	2.33 (1.88–2.89)	2.09 (1.67–2.62)	Overestimated	1.86 (1.52–2.28)	1.86 (1.51–2.28)	2.20 (1.77–2.74)
Fasting	About right	1.00	1.00	1.00	Accurate	1.00	1.00	1.00
	Underweight	1.23 (0.95–1.60)	1.17 (0.90–1.52)	1.29 (0.97–1.71)	Underestimated	1.66 (1.23–2.23)	1.62 (1.20–2.19)	1.49 (1.10–2.02)
	Overweight	2.67 (2.20–3.25)	2.60 (2.13–3.16)	2.39 (1.94–2.94)	Overestimated	1.83 (1.53–2.20)	1.80 (1.50–2.17)	2.16 (1.77–2.63)

Model 1 adjusted for socio-demographic characteristics of age, gender, location of school, paternal education and maternal education; model 2 adjusted for model 1 plus lifestyle behaviors of current smoking, breakfast consumption and muscle strengthening activity; model 3 adjusted for model 2 plus body mass index.

## Discussion

Based on 17,359 school-aged children and adolescents from Zhejiang Province in China, this study was conducted to describe the self-perceived weight status and accuracy of weight perceptions, and examine their relationships with weight control related behaviors. To our knowledge, this is one of few studies attempting to link the weight perceptions to various weight control behaviors among Chinese youth.

Overall, we suggested that about a third of children and adolescents (34.19%) perceived themselves as overweight, and 23.01% as underweight. To some extent, these results were comparable to findings from other Chinese studies. For example, a regional study conducted in Hong Kong revealed that among 1132 secondary school students, 35.7% had a perception of overweight, while 20.5% had a underweight perception, approaching our total estimates [21]. Another regional study from two provinces of Shandong and Qinghai suggested that the percentage of perceiving themselves as overweight or obese and underweight among 3rd and 7th grade studentswas 26.46% and 26.02%, respectively [15]. More recently, a national survey of high school and college students showed that 22.8% perceived themselves as overweight or obese and 27.6% as underweight [22]. Worldwide, in the United States, the proportion of students in grades 9–12 with a perception of overweight and underweight was reported to be 28.3% and 24.5%, respectively [23]. In South Korea, survey results from 8th and 11th grades students showed that 24.8% perceived themselves as being overweight and 20.5% being too thin [24]. Furthermore, in Brazil, one study found that 39.6% of the adolescents aged 15–19 years perceived themselves as above the expected body weight and 29.3% as below [20].

Regarding the accuracy of weight perceptions, our results showed that 45.44% of children and adolescents have misperceived their actual weight status, with 35.54% overestimation and 9.90% underestimation. Consistently, the aforementioned Chinese study in Shandong and Qinghai provinces suggested that the total prevalence of weight misperception among children and adolescents was 44.50% [15]. Hsu et al. reported that the prevalence of body weight misperception was 43.2% (26.4% overestimation and 16.8% underestimation) among Taiwan teenagers [25]. Representative data from 2004–09 China Health and Nutrition Survey (CHNS) also confirmed that more than 40% of the Chinese youth inaccurately estimated their weight status (45.5% for children 6–11 and 39.4% for adolescents 12–17 years of age) [14]. Globally, although the international trend in misperceived weight in youth is uncertain over time [26, 27], considerable studies have suggested that the weight misperception is prevalent among adolescents. With a nationally representative sample of 8th graders from the Early Childhood Longitudinal Study-Kindergarten Class (ECLS-K), authors updated that 42.1% of US adolescents misperceived their weight status (35.3% underestimation and 6.8% overestimation) [10]. Data from another national survey in South Korea, consisting of 72,339 adolescents aged 12–18 years, showed that over half of the participants (23.4% underestimation and 26.8% overestimation) had a misperception of their own weight status [11]. The variation in the prevalence of self-weight perception and misperception among youth across the countries and regions may be due to the differences in participant characteristics, weight status measurement, sample size, and study period. Particularly, consistent with previous findings indicating the gender difference in weight perceptions [15, 28, 29], we observed a larger proportion of self-perceived overweight (41.27% vs. 27.01%) and overestimation perception (48.42% vs. 22.48%) in girls, while the underestimation was the dominant misperception in boys (15.53% vs. 4.35%). Besides, the relatively high prevalence of weight misperception among normal weight adolescents (50.46%) reported in this study also supported the proposed conception that misperception is a common problem for chlidren and adolescents in all weight categories [10].

Meanwhile, this study focused on examining the associations of self-perceived weight status and misperception with weight control related behaviors. Consistent with numerous cross-sectional studies [30–33], we found that the self-perception of being overweight among children and adolescents was positively associated with weight control attempt and specific health or unhealthy weight control behaviors, after adjustment for potential confounders. Worldwide, a cross-national study conducted in over 30 countries suggested that the self-perception of overweight was the most important factor leading to attempt to lose weight among adolescents [34]. Furthermore, findings from the National Longitudinal Study of Adolescents Health (Add Health) provided more convincing evidence that the self-perception of being overweight was significantly associated with unhealthy weight control behaviors, such as purging and diet pill use [35]. At present, the possible mechanisms underlying the positive associations between overweight perception and unhealthy weight control behaviors among children and adolescents are complicated and uncertain. However, previous studies supported the role of negative body imagine in the development of unhealthy weight control behaviors [36, 37]. More importantly, in line with previous reports [31, 38], our results suggested that children and adolescents’ weight misperception was significantly associated with weight control attempt and specific behaviors compared to accurate perception, irrespective of actual weight status. Specifically, in expected ways, weight overestimating adolescents in this study were more likely to try to control weight and engage in weight control behaviors. Despite a direct comparison to our results was impossible, it seemed that among South Korean adolescents, overestimation misperception was associated with greater likelihood of various weight control practices, including exercising, fasting, as well as laxative use and self-induced vomiting [13, 39]. Similarly, an US study of normal weight adolescents also linked weight overestimation to unhealthy weight control behaviors [12]. Our results supported the previous findings to some extent, that children and adolescents overestimating body weight reported a higher body dissatisfaction, a drive for thinness and lower self-esteem [40]. By contrast, there is little discussion on the association between underestimation misperception and weight control behaviors in the literature. In this study, we observed that weight underestimation was positively associated with taking laxatives, diet pills, and fasting, which were consistent with limited reports from South Korea [39] and Taiwan [25]. These findings suggested that there were important implications to improve weight perceptions and reduce misperceptions for addressing the unhealthy weight control behaviors among children and adolescents. Future prospective studies are needed to understand the causality of these relationships, and the research on possible mechanisms is clearly warranted.

The present study had several strengths. This study was one of the few studies examining the associations between weight perceptions and weight control related behaviors in mainland China. It was a representative ongoing school-based study with relatively large sample of 17,359 adolescents in Zhejiang Province. However, our study had some limitations. Firstly, due to the cross-sectional nature of the research, causality of the reported linkages between the exposure and outcome variables cannot be inferred from this study. Secondly, data of height and weight among children and adolescents were collected via self-administered questionnaires. Although self-reported data are widely used in previous surveys, according to the relevant validation studies, children and adolescents with self-reported height and weight have been found to underestimate the prevalence of overweight / obesity and weight misperception [41, 42].

## Conclusions

In conclusion, our study suggested that over a third of Chinese children and adolescents perceived themselves as being overweight and nearly half have misperceived their weight status. Self-perceived overweight and weight misperception were found to be associated with weight control attempt and specific weight control behaviors. With the increasing prevalence of childhood and adolescence obesity, the educational programs on weight control strategies should highlight the educational component of weight perceptions.

## Supporting information

S1 File (DOCX)

S1 Dataset (CSV)
