# Supplementary material for: Relationships of weight perceptions with weight control related behaviors among Chinese children and adolescents: A school-based study in Zhejiang Province
Source: PLoS One. 2023 May 17;18(5):e0285205. doi: 10.1371/journal.pone.0285205 (PMC10191311; doi:10.1371/journal.pone.0285205)
Supplement: S1 File — (DOCX) [file pone.0285205.s001.docx]

| Supplementary Table 1. Adjusted odds ratios (95% CIs) of weight control related behaviors by self-perception weight status within subgroups defined by gender, location of school and body mass index. | | | | | | | | | | | |
| --- | --- | --- | --- | --- | --- | --- | --- | --- | --- | --- | --- |
| Outcomes | Self-perception weight status | Gender | | Heterogeneity test: χ2 (P) | Location of school | | Heterogeneity test: χ2 (P) | Body mass index | | | Heterogeneity test: χ2 (P) |
|  |  | Boys | Girls |  | Rural | Urban |  | Underweight | Normal weight | Overweight  /obesity |  |
| Trying to control weight | About right | 1.00 | 1.00 | NA | 1.00 | 1.00 | NA | 1.00 | 1.00 | 1.00 | NA |
|  | Underweight | 0.51 (0.45-0.58) | 0.41 (0.35-0.47) | 4.84 (0.03) | 0.46 (0.41-0.52) | 0.45 (0.39-0.52) | 0.05 (0.82) | 0.50 (0.43-0.58) | 0.44 (0.39-0.50) | 0.30 (0.16-0.54) | 3.58 (0.17) |
|  | Overweight | 3.45 (3.01-3.97) | 2.10 (1.88-2.34) | 30.41 (<0.001) | 2.42 (2.17-2.70) | 2.91 (2.53-3.33) | 4.24 (0.04) | 3.67 (2.68-5.03) | 2.43 (2.22-2.67) | 2.71 (1.81-4.07) | 6.20 (0.05) |
| Healthy weight control behavior |  |  |  |  |  |  |  |  |  |  |  |
| Exercising | About right | 1.00 | 1.00 | NA | 1.00 | 1.00 | NA | 1.00 | 1.00 | 1.00 | NA |
|  | Underweight | 0.37 (0.33-0.43) | 0.39 (0.34-0.46) | 0.26 (0.61) | 0.34 (0.29-0.38) | 0.40 (0.34-0.46) | 2.47 (0.12) | 0.40 (0.34-0.47) | 0.36 (0.31-0.41) | 0.31 (0.16-0.59) | 1.26 (0.53) |
|  | Overweight | 3.66 (3.18-4.21) | 1.99 (1.78-2.21) | 45.44 (<0.001) | 2.31 (2.07-2.57) | 2.80 (2.44-3.21) | 4.66 (0.03) | 3.01 (2.23-4.05) | 2.35 (2.15-2.58) | 3.94 (2.64-5.87) | 8.06 (0.02) |
| Unhealthy weight control behaviors |  |  |  |  |  |  |  |  |  |  |  |
| Dieting | About right | 1.00 | 1.00 | NA | 1.00 | 1.00 | NA | 1.00 | 1.00 | 1.00 | NA |
|  | Underweight | 0.43 (0.34-0.54) | 0.42 (0.34-0.51) | 0.02 (0.88) | 0.39 (0.32-0.47) | 0.40 (0.31-0.51) | 0.02 (0.88) | 0.32 (0.25-0.40) | 0.52 (0.43-0.64) | 0.37 (0.12-1.17) | 9.61 (0.01) |
|  | Overweight | 4.06 (3.48-4.73) | 2.35 (2.11-2.62) | 32.57 (<0.001) | 2.87 (2.56-3.22) | 2.81 (2.44-3.23) | 0.05 (0.82) | 3.09 (2.28-4.20) | 2.73 (2.48-3.01) | 6.63 (3.83-11.47) | 10.10 (0.01) |
| Taking laxatives | About right | 1.00 | 1.00 | NA | 1.00 | 1.00 | NA | 1.00 | 1.00 | 1.00 | NA |
|  | Underweight | 2.32 (1.47-3.65) | 1.78 (1.04-3.04) | 0.55 (0.46) | 1.94 (1.28-2.93) | 2.88 (1.54-5.36) | 1.07 (0.30) | 1.09 (0.61-1.96) | 3.13 (2.07-4.73) | 2.48 (0.54-11.44) | 8.38 (0.02) |
|  | Overweight | 1.36 (0.80-2.29) | 2.44 (1.70-3.52) | 3.21 (0.07) | 1.62 (1.14-2.30) | 3.10 (1.86-5.19) | 4.19 (0.04) | 3.15 (1.46-6.78) | 2.22 (1.61-3.07) | 0.68 (0.19-2.41) | 4.12 (0.13) |
| Taking diet pills | About right | 1.00 | 1.00 | NA | 1.00 | 1.00 | NA | 1.00 | 1.00 | 1.00 | NA |
|  | Underweight | 1.95 (1.21-3.14) | 0.60 (0.35-1.04) | 10.19 (0.001) | 1.10 (0.74-1.64) | 1.27 (0.72-2.26) | 0.16 (0.69) | 0.48 (0.28-0.83) | 1.81 (1.20-2.73) | 2.52 (0.55-11.43) | 15.64 (<0.001) |
|  | Overweight | 2.62 (1.64-4.18) | 1.88 (1.45-2.44) | 1.48 (0.22) | 1.94 (1.46-2.58) | 2.37 (1.63-3.43) | 0.70 (0.40) | 2.03 (1.02-4.05) | 2.25 (1.75-2.89) | 1.65 (0.49-5.53) | 0.30 (0.86) |
| Fasting | About right | 1.00 | 1.00 | NA | 1.00 | 1.00 | NA | 1.00 | 1.00 | 1.00 | NA |
|  | Underweight | 2.16 (1.43-3.26) | 0.85 (0.56-1.30) | 9.63 (0.002) | 1.44 (1.01-2.06) | 1.04 (0.64-1.67) | 1.14 (0.29) | 0.73 (0.54-1.18) | 2.06 (1.46-2.90) | 3.55 (0.62-20.43) | 16.50 (<0.001) |
|  | Overweight | 2.97 (2.02-4.37) | 2.17 (1.69-2.78) | 1.79 (0.18) | 2.39 (1.82-3.13) | 2.40 (1.73-3.33) | 0.00 (0.99) | 3.73 (2.16-6.43) | 2.33 (1.84-2.93) | 4.15 (0.98-17.54) | 2.88 (0.24) |
| All models were stratified by gender, location of school and body mass index, and adjusted for age, gender, location of school, paternal education, maternal education, current smoking, breakfast consumption, muscle strengthening activity, and body mass index, except for the stratification variable itself. | | | | | | | | | | | |

| Supplementary Table 2. Adjusted odds ratios (95% CIs) of weight control related behaviors by accuracy of weight perceptions within subgroups defined by gender, location of school and body mass index. | | | | | | | | | | | |
| --- | --- | --- | --- | --- | --- | --- | --- | --- | --- | --- | --- |
| Outcomes | Weight perceptions | Gender | | Heterogeneity test: χ2 (P) | Location of school | | Heterogeneity test: χ2 (P) | Body mass index | | | Heterogeneity test: χ2 (P) |
|  |  | Boys | Girls |  | Rural | Urban |  | Underweight | Normal weight | Overweight  /obesity |  |
| Trying to control weight | Accurate | 1.00 | 1.00 | NA | 1.00 | 1.00 | NA | 1.00 | 1.00 | 1.00 | NA |
|  | Underestimated | 0.43 (0.37-0.49) | 0.36 (0.28-0.45) | 1.60 (0.21) | 0.38 (0.32-0.44) | 0.43 (0.35-0.52) | 0.91 (0.34) | NA | 0.44 (0.39-0.50) | 0.24 (0.17-0.33) | 11.25 (0.001) |
|  | Overestimated | 2.78 (2.46-3.13) | 2.13 (1.93-2.35) | 11.26 (0.001) | 2.22 (2.02-2.45) | 2.70 (2.39-3.06) | 5.99 (0.01) | 2.37 (2.06-2.73) | 2.43 (2.22-2.67) | NA | 0.08 (0.77) |
| Healthy weight control behavior |  |  |  |  |  |  |  |  |  |  |  |
| Exercising | Accurate | 1.00 | 1.00 | NA | 1.00 | 1.00 | NA | 1.00 | 1.00 | 1.00 | NA |
|  | Underestimated | 0.32 (0.27-0.37) | 0.37 (0.29-0.47) | 0.97 (0.32) | 0.30 (0.25-0.35) | 0.38 (0.31-0.46) | 3.19 (0.07) | NA | 0.36 (0.31-0.41) | 0.17 (0.12-0.24) | 15.49 (<0.001) |
|  | Overestimated | 3.24 (2.85-3.68) | 2.08 (1.88-2.29) | 28.95 (<0.001) | 2.37 (2.14-2.61) | 2.73 (2.41-3.10) | 2.99 (0.08) | 2.92 (2.51-3.41) | 2.35 (2.15-2.58) | NA | 5.70 (0.02) |
| Unhealthy weight control behaviors |  |  |  |  |  |  |  |  |  |  |  |
| Dieting | Accurate | 1.00 | 1.00 | NA | 1.00 | 1.00 | NA | 1.00 | 1.00 | 1.00 | NA |
|  | Underestimated | 0.38 (0.30-0.49) | 0.44 (0.33-0.60) | 0.55 (0.46) | 0.36 (0.28-0.46) | 0.40 (0.30-0.54) | 0.29 (0.59) | NA | 0.52 (0.43-0.64) | 0.11 (0.07-0.18) | 35.31 (<0.001) |
|  | Overestimated | 3.79 (3.26-4.41) | 2.42 (2.18-2.69) | 22.82 (<0.001) | 2.86 (2.56-3.20) | 2.82 (2.45-3.23) | 0.02 (0.88) | 3.86 (3.08-4.83) | 2.73 (2.48-3.01) | NA | 7.68 (0.01) |
| Taking laxatives | Accurate | 1.00 | 1.00 | NA | 1.00 | 1.00 | NA | 1.00 | 1.00 | 1.00 | NA |
|  | Underestimated | 2.72 (1.75-4.24) | 2.20 (1.14-4.23) | 0.28 (0.60) | 2.24 (1.45-3.45) | 3.48 (1.91-6.35) | 1.36 (0.24) | NA | 3.13 (2.07-4.73) | 2.34 (0.97-5.61) | 0.35 (0.56) |
|  | Overestimated | 1.40 (0.87-2.23) | 2.07 (1.48-2.90) | 1.76 (0.19) | 1.57 (1.13-2.18) | 2.44 (1.54-3.86) | 2.34 (0.13) | 1.21 (0.72-2.03) | 2.22 (1.61-3.07) | NA | 3.80 (0.05) |
| Taking diet pills | Accurate | 1.00 | 1.00 | NA | 1.00 | 1.00 | NA | 1.00 | 1.00 | 1.00 | NA |
|  | Underestimated | 2.05 (1.30-3.24) | 0.98 (0.51-1.88) | 3.30 (0.07) | 1.62 (1.06-2.48) | 1.23 (0.65-2.33) | 0.50 (0.48) | NA | 1.81 (1.20-2.73) | 0.96 (0.43-2.14) | 1.90 (0.17) |
|  | Overestimated | 2.43 (1.61-3.66) | 2.10 (1.62-2.73) | 0.35 (0.56) | 2.23 (1.68-2.95) | 2.14 (1.50-3.04) | 0.03 (0.86) | 2.38 (1.42-3.98) | 2.25 (1.75-2.89) | NA | 0.04 (0.85) |
| Fasting | Accurate | 1.00 | 1.00 | NA | 1.00 | 1.00 | NA | 1.00 | 1.00 | 1.00 | NA |
|  | Underestimated | 1.77 (1.21-2.57) | 1.14 (0.64-2.05) | 1.55 (0.21) | 1.45 (0.99-2.12) | 1.54 (0.94-2.55) | 0.04 (0.85) | NA | 2.06 (1.46-2.90) | 0.46 (0.19-1.11) | 9.63 (0.002) |
|  | Overestimated | 2.40 (1.69-3.40) | 1.96 (1.54-2.49) | 0.88 (0.35) | 2.09 (1.62-2.69) | 2.29 (1.67-3.14) | 0.20 (0.66) | 1.92 (1.25-2.95) | 2.33 (1.84-2.93) | NA | 0.60 (0.44) |
| All models were stratified by gender, location of school and body mass index, and adjusted for age, gender, location of school, paternal education, maternal education, current smoking, breakfast consumption, muscle strengthening activity, and body mass index, except for the stratification variable itself. | | | | | | | | | | | |
